# Black P/graphene hybrid: A fast response humidity sensor with good reversibility and stability

**DOI:** 10.1038/s41598-017-10848-3

**Published:** 2017-09-05

**Authors:** Duy-Thach Phan, Inyong Park, Ah-Ram Park, Cheol-Min Park, Ki-Joon Jeon

**Affiliations:** 1 Department of Environmental Engineering, Inha University, 100 Inha-ro, Nam-gu, Incheon 22212 Republic of Korea; 2 School of Materials Science and Engineering, Kumoh National Institute of Technology, Gumi, Gyeongbuk 39177 Republic of Korea

## Abstract

Black phosphorus (BP) materials have attracted considerable attention owing to their ultra-sensitive humidity sensing characteristics because of the natural absorption of water (H_2_O) molecules on the BP surface caused by the specific 2D layer-crystalline structure. On the other hand, the BP-based humidity sensor is less repeatable due to the instability of BP with water molecules and the stability of the sensor is reduced. In this study, this limitation of the BP-based humidity sensor was overcome by preparing a BP/graphene hybrid as a novel humidity sensing nanostructure. The BP/graphene interface improved the stability of the humidity sensor after a few weeks with a linear response within the relative humidity (RH) range of 15–70%. The sensor’s response/recovery speed of the humidity sensor was extremely fast within few seconds. The response (S) of the humidity sensor based on the BP/graphene hybrid is 43.4% at RH = 70%. The estimated response and recovery time of the sensor is only 9 and 30 seconds at RH = 70% at room temperature. The experimental investigation reveals that the BP/graphene hybrid not only improves the reversibility and hysteresis factors but also enhances the stability of the humidity sensor.

## Introduction

Humidity sensing techniques have been investigated in many areas such as the industrial processing, environment, food, agricultural, and indoor applications^1^. Various humidity sensor technologies have been developed, such as those based on resistive^1^, semiconductor^1^, optical^2^, and surface acoustic wave technologies^3^. Among these devices, resistive sensors have several advantages compare with other types such as simple structure, high sensitivity, rapid response, low cost and low power consumption^1^. Popular humidity sensing materials include polymers^4^, semiconducting metal oxides^1^, and carbon-based materials (carbon nanotube and graphene oxide)^5^. These conventional materials with a range of nanostructures offer many advantages for use in humidity sensing. On the other hand, humidity sensors from these conventional nanomaterials still exhibit a slow response/recovery time and in some cases require heating temperatures for operation^1–5^. Recently, new two dimension (2D) nanomaterials have demonstrated many advantages in the sensing area with a planar crystallite structure, large surface-to-volume, and low electronic noise^6^. The unique properties of new 2D materials, such as graphene^5–7^, black phosphorus (BP)^8–10^, and tin disulfide (SnS_2_)^11^, provided superior sensing factors including single molecules agents, ultra-fast super sensitivity and low power consumption.

BP has been reported as a novel material for gas and humidity sensing with excellent properties^12, 13^. In particular, BP is a new excellent humidity sensing material with ultra-sensitivity and a rapid response^12^. However, BP has low stability and reacts naturally with water molecules, limiting its long-term stability in humid environment^12^. To overcome this limitation, there have been some attempts to passivate the surface of BP^14^, encapsulate it with graphene^15^, or use a composite/hybrid with other materials^16–18^. In particular, chemical bonding between phosphorus and carbon (P-C) has been reported to play a significant role in stabilizing BP/graphene composites/hybrids for practical applications^15–17^. On the other hand, the conventional composites/hybrids are usually limited due to defect formation at the interface, leading to lower stability of the electronic device. Graphene-based heterojunction presents an interesting alternative way to overcome this limitation because of excellent properties of graphene as a support material. In addition, the combination of these new 2D materials on graphene to form heterojunctions between 2D materials has advantages in 2D electronic device such as low electronic noise, low power consumption, and good stability at the interfaces^6, 15^. The new 2D material and graphene heterojunction control provides the latest advantages of 2D electronics, such as low electronic noise, low power consumption and excellent stability interfaces.

To date, exfoliated 2D layers, particularly BP, could only be transferred in small sizes onto substrates in basic and proof-of-concept studies^12, 13^. However, to take advantage of unique characteristics of scalable technologies, it is essential to establish a mass production process for 2D materials synthesis and sensor device fabrication. This very exciting challenge will be addressed in the current work. In this study, a large quantity of BP materials was synthesized in powder form (several gram-scale) with a novel technique of high energy ball milling (HEBM). The BP powder was exfoliated by a mild ultrasonication process and BP/graphene heterojunction was formed on the graphene surface using an electrospray system for humidity sensor. The humidity sensing performances of the sensors prepared with a pure BP and BP-graphene heterojunction were investigated and compared in terms of the sensitivity, reversibility, and stability. The role of graphene and the interface between BP/graphene for humidity sensors was investigated in detail.

## Results and Discussion

**Figure 1:**
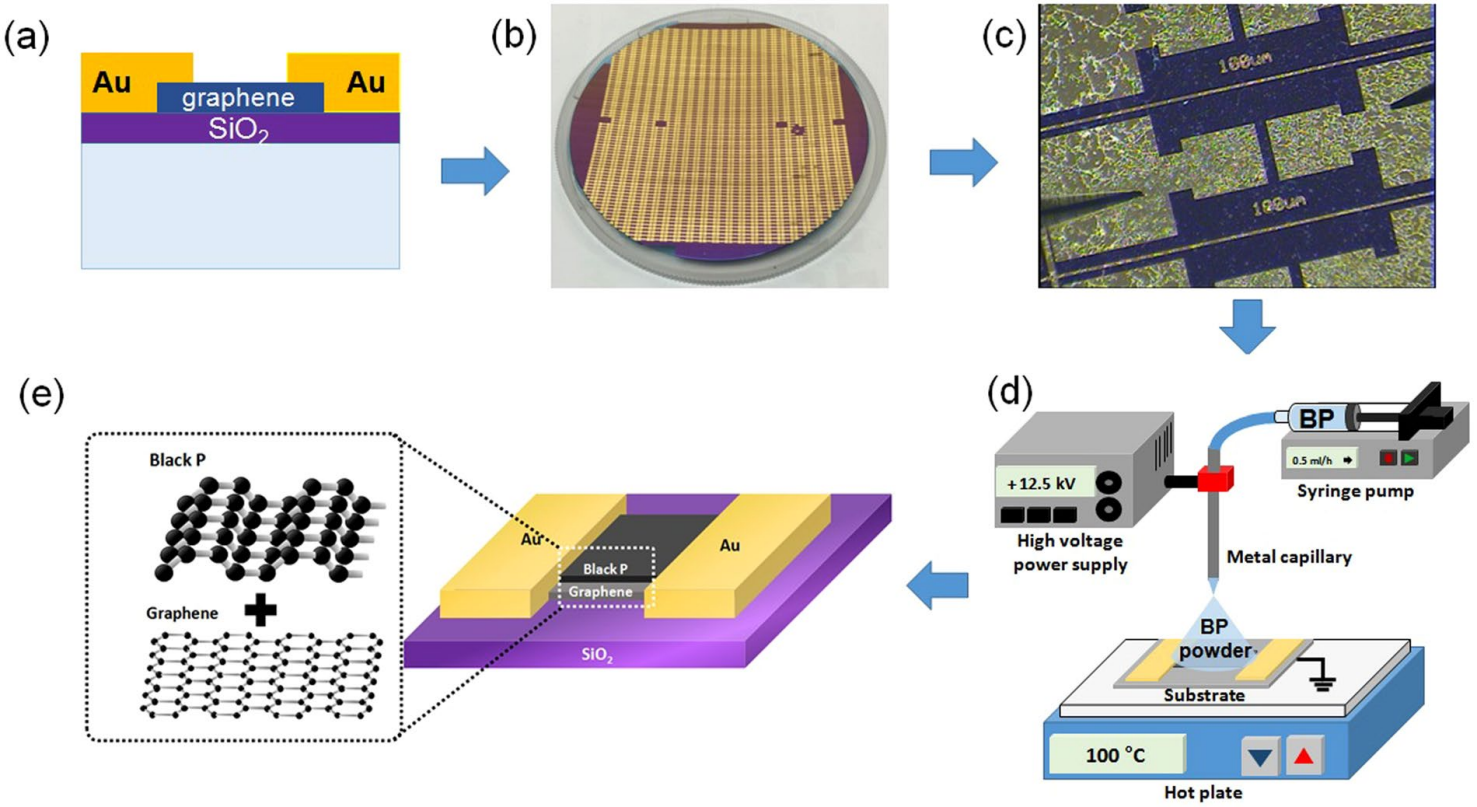
Sequence of sensor fabrication process of humidity sensor using BP/graphene heterojunction (**a**) schematic diagram of the sensor, (**b**) image of the full wafer of fabricated sensor, (**c**) optical image of single graphene chip, (**d**) electrospray system to deposit BP on graphene, and (**e**) schematic diagram of the sensor based on the BP/graphene heterojunction.

**Figure 2:**
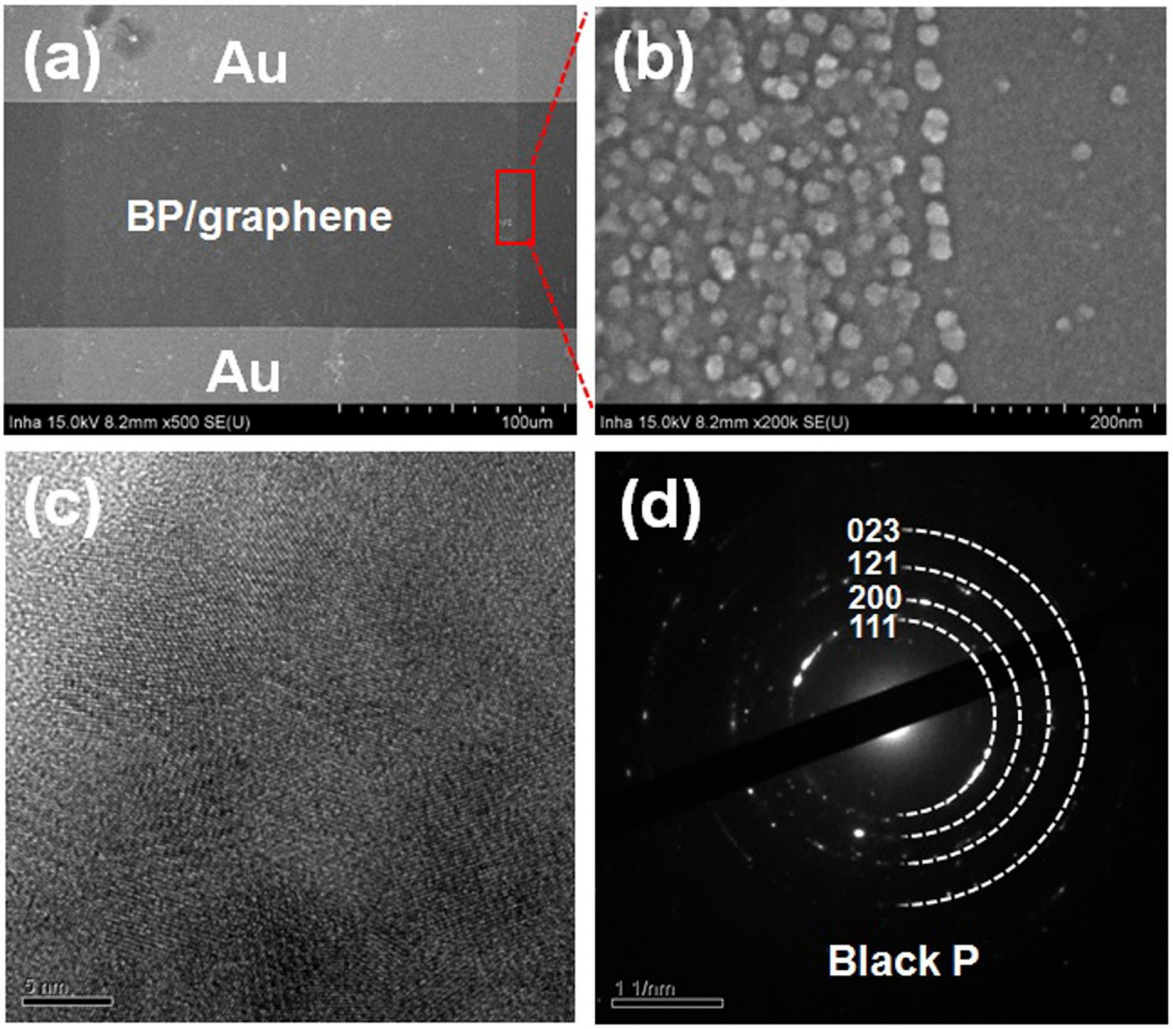
SEM images (**a**,**b**) humidity sensor of BP/graphene, (**c**) TEM images and (**d**) SAED of BP flakes.

**Figure 3:**
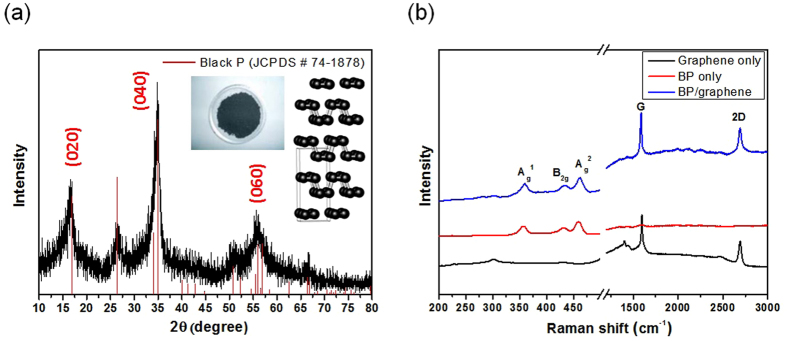
Crystallite properties, (**a**) XRD pattern of BP and (**b**) Raman spectra of BP only, pure graphene and BP/graphene heterojunction.

Figure 1 presents the sequence of humidity sensor fabrication process using BP material on graphene. The fabrication process starts at transfering graphene onto SiO_2_/Si with wafer-level. Then, the platform of humidity sensor chip comprise patterned graphene between two gold (Au) electrode. The BP powder was synthesized by a HEBM method and deposited on the patterned graphene by electro-spray (see in Supplementary data for more detail). Figure 2a presents a SEM image of fabricated humidity sensor based on BP/graphene hybrid. The patterned graphene was located between two gold (Au) electrodes. The distance between two Au-electrodes was 100 µm. The BP particles were synthesized using a commercially available red P powder according to the HEBM technique (Fig. 2b). The size of BP particles was ca. 200 nm, which were well deposited on the patterned graphene area by an electrospray system, as shown in Fig. 2b.The well-developed crystalline structured BP with an interesting 2D puckered-layer crystalline structure was confirmed by HRTEM (Fig. 2c). HRTEM electron diffraction, as in Fig. 2d showed that the sample was corresponded well to the orthorhombic BP. Figure 3a shows the XRD pattern of BP powder. Major peaks in the pure BP sample were observed at 2θ = 16.44°, 34.8°, and 55.78°, which corresponds to the (020), (040), and (060) planes of BP, respectively, as denoted by the International Center for Diffraction Data (JCPDS # 74–1878). Figure 3b presents the Raman spectrum for the BP-graphene heterojunction, where the characteristic A_g_^1^, B_2g_, and A_g_^2^ peaks of BP are clearly visible at 359, 432, and 462 cm^−1^, respectively^8, 19^. The A_g_^1^ and A_g_^2^ peaks of the pure BP sample were observed at 356 and 459 cm^−1^, respectively. The Raman spectrum of single layer graphene consisted of two major sharp peaks (G and 2D peaks) at 1584 and 2674 cm^−1^. The major peaks (G and 2D peaks) originated from the doubly degenerate zone center E_2g_ mode and the second order zone boundary phonons, respectively^20^. The D peak at 1350 cm^−1^ is related to the defects present^20^. The features of those peaks are indicators of the quality of graphene, such as doping and strain. The G and 2D peaks in BP/graphene were 1590 and 2689 cm^−1^, respectively. No obvious increase in the D peak in the Raman spectra before and after depositing BP powder on graphene was observed. The as-synthesized single layer graphene has Raman spectra with 2D/G ratio > 1. However, the 2D/G ratio value was reduced (2D/G < 1) after the sensor fabrication process (see detail in Fig. S4, Supplementary data).

**Figure 4:**
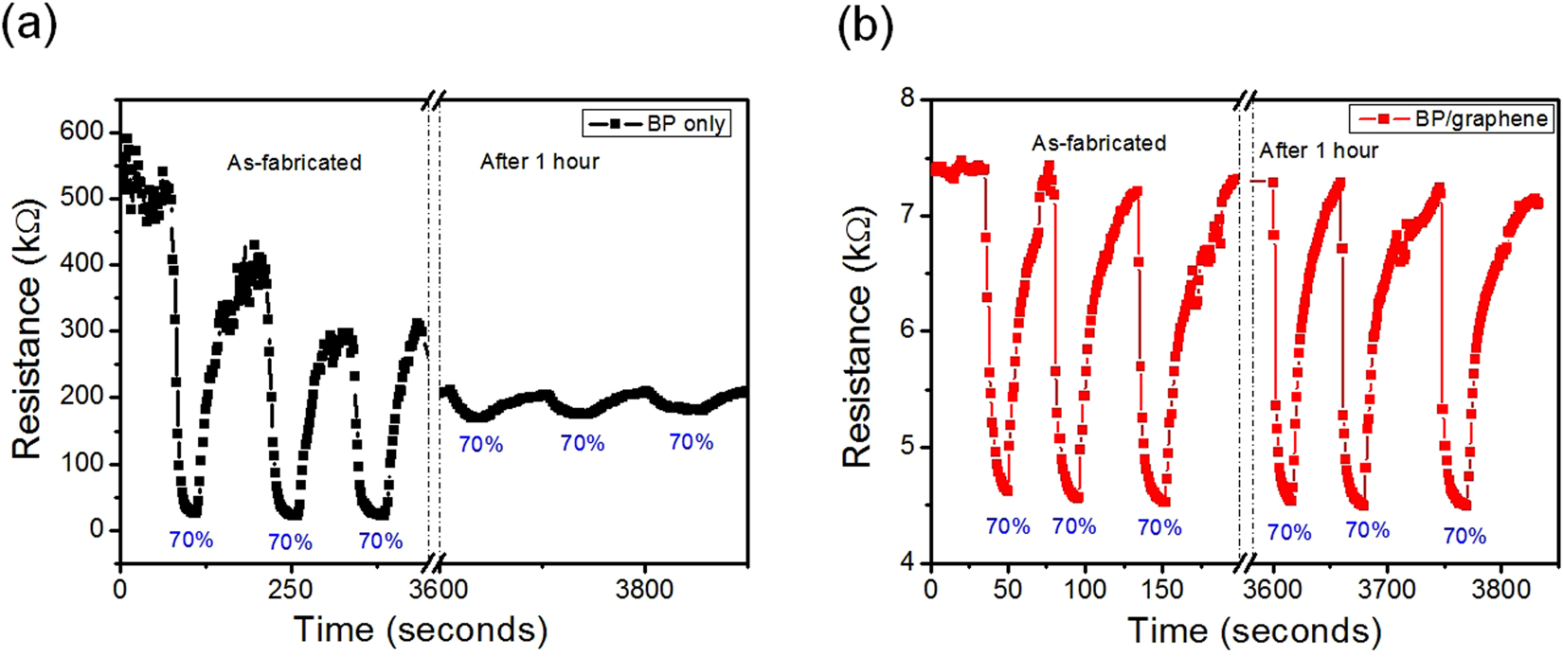
Transient response and estimated stability of the humidity sensor after 1 hour based on (**a**) BP only and (**b**) BP/graphene heterojunction.

Figure 4 shows the transient response of the as-fabricated humidity sensor, which is based on a pure BP and BP-graphene heterojunction, and their response after 1 hour with a reproducible relative humidity (RH) of 70%. Figure 4a shows the response of humidity sensor using pure BP powder with non-repeatability and degradation after 1 hour. This degradation of the BP-based sensor in a high humidity environment was similar to other works^21, 22^, and were reported in other published papers^21, 22^. Compared to the pure BP sample, the humidity sensor based on BP-graphene in Fig. 4b showed a much better response with the advantages of fast response/recovery, good repeatability and non-degradation after 1 hour. For comparison, the response of as-fabricated humidity sensor using pure graphene can be found in Fig. S5 (Supplementary data). The initial resistance of the humidity sensor was 7.5 kΩ and 500 kΩ for the BP/graphene and pure BP sample, respectively. Owing to the good conductivity of single layer graphene (the initial resistance of humidity sensor using pure graphene was only 0.63 kΩ, see in Fig. S5, Supplementary data), the BP/graphene heterojunction sensor had a very low resistance compared to that of pure BP. Moreover, the signal-to-noise level in the BP/graphene heterojunction was higher than that of pure BP, leading to a clear signal of resistivity sensor in the BP/graphene sample.

**Figure 5:**
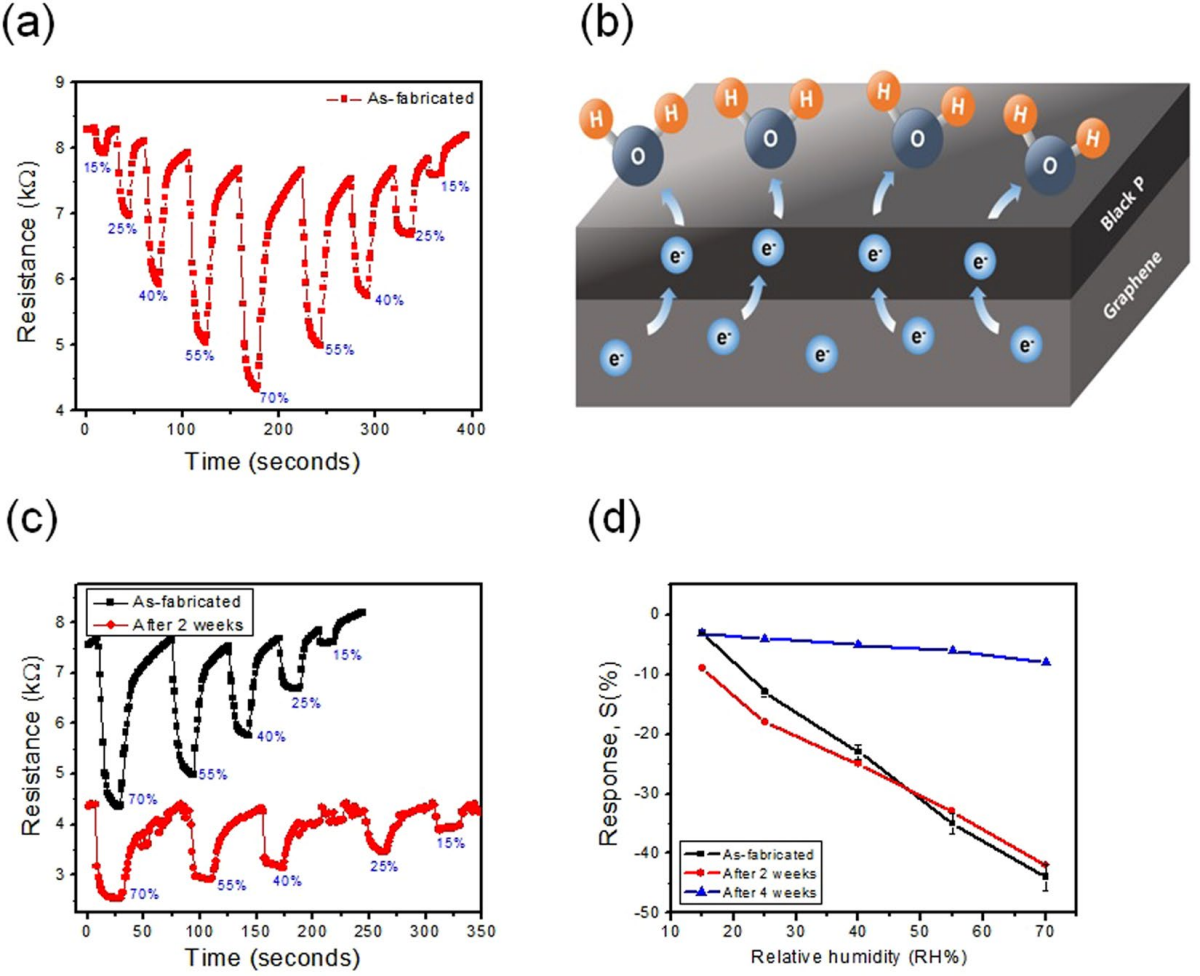
Humidity sensor properties, (**a**) Transient response of the humidity sensor using a BP/graphene heterojunction with various RH level, (**b**) humidity sensing mechanism, (**c**) transient response of the humidity sensor as-fabricated and after 2 weeks and (**d**) linearity of the sensors.

The sensor relative response (S) is defined as the percentage resistance change of the resistivity sensor by exposure to humidity:

1 $${\rm{S}}( \% )={\rm{\Delta }}{{\rm{R}}/{\rm{R}}}_{{\rm{a}}}\times 100=({{\rm{R}}}_{{\rm{h}}}-{{\rm{R}}}_{{\rm{a}}})/{{\rm{R}}}_{{\rm{a}}}\times 100$$

where R_a_ is the resistance of the sensors in the presence of dry N_2_ gas only and R_h_ is the resistance in the presence of humidity at the given concentrations. The response time is defined as the time required for the humidity sensor to reach 90% of the resistance change (ΔR) when the sensor is exposed to a given humidity. The recovery time is defined as the time needed to recover to 90% of the initial baseline after turning-off the humidity. The responses S (%) of the humidity sensor based on BP-graphene heterojunction calculating from Fig. 5a were 43.4, 35, 25.1, 13, and 3% with a RH of 70, 55, 40, 25, and 15%, respectively. The response/recovery time of humidity sensor was 9/30 seconds at a RH of 70%.Compared to other similar studies, the humidity sensor using the liquid exfoliation of pure BP has a response/recovery time of 255/10 seconds^21^ and 5/5 seconds^12^. Meanwhile, the response/recovery time of our humidity sensor using pure BP was 24/72 seconds. The results showed that the humidity sensor using BP/graphene heterojunction is better than that using pure BP with a 2-fold faster response time. Moreover, the humidity sensor using pure BP without a passivation method showed large degradation after a few cycles (within 2 hours)^21^. Fig. 5b explains the sensing mechanism of the humidity sensors based on the BP/graphene heterojunction. The humidity (H_2_O) molecules withdraw a free electron from the BP flakes and increase the hole density in BP. The BP exhibits a p-type semiconductor behavior^8, 9, 15^. Therefore, the increasing hole density in p-type BP leads to decreasing resistance in the humidity sensor of the pure BP sample (as see in Fig. 4a). With BP-graphene heterojunction sample, this encourages the free electron to graphene transfer to BP via the BP/graphene interface. Finally, the increasing holes in p-type graphene decrease the resistance of the BP/graphene sample (as see in Fig. 4b).

Figure 5c shows the transient response of the humidity sensor based on the as-fabricated BP-graphene heterojunction and the response on the same sensor sample after 2 weeks. In general, there is little difference between these responses, except that the response/recovery is slower after 2 weeks. On the other hand, the sensor had a large reduction in response after 4 weeks, as shown in Fig. 5d (see also in the Supplementary data). Figure 5d confirmed the linearity of humidity sensor in the RH range of 15–70% and the stability of the sensor after 2 weeks. Compared to previous publications on humidity sensing using BP materials^12^, the BP-graphene heterojunction humidity sensor in this study has the advantageous features of a fast response/recovery time, linearity and mass production process of sensor. The degradation of the BP only sensor was caused by lower stability of the surface of the BP flake in moisture and humidity^14^. In this case, pure BP played a role both as a humidity sensing material and conducting patch between the Au electrodes in the sensor device, leading to higher noise and degradation during humidity absorption/desorption. The sensing mechanism of BP/graphene heterojunction involves charge transfer at the interface between BP/graphene and graphene playing a role as a stable conducting path. In addition, due to the good stability at the BP/graphene interface^15–17^, the humidity sensor could show better stability and reversibility, compared to the sensor using the BP only sample. Passivation of BP surface is essential for sensing applications^12, 22^, and electronic devices^14, 15^ based on BP materials. This is the greatest challenge for applying BP as a sensing material because the passivated BP process reduced the sensitivity of the BP surface, leading to the complicated fabrication of the sensor. However, the sensor based on passivated BP showed a long-term stability up to one month^22^ or three months^12^. In this study, the BP/graphene heterojunction humidity sensor showed high sensitivity, fast/response recovery owing to BP without a passivation process and acceptable stability for up to two-weeks, which is better than that of the sensor using the pure exfoliated BP material with stability for only a few hours.

## Conclusions

A humidity sensor based on BP/graphene heterojunction was developed using a large scale fabrication technique. The role of graphene in the heterojunction of the humidity sensor was investigated by comparing the sensor performance of the BP and BP/graphene sample. The BP-only samples showed good sensitivity to humidity with the advantages of a strong response and rapid response/recovery time, but were less stable, non-linear and reversible. On the other hand, the BP/graphene heterojunction-based humidity sensor overcomes these limitations and has excellent sensing properties with a response of 43.4% and a response/recovery time of 9/30 seconds with a RH of 70%. In addition, the humidity sensor has good repeatability, low hysteresis and long-term stability over two weeks by forming a BP-graphene heterojunction. Moreover, it solves the instability of the BP-only sensor.

## Methods

The BP powder was synthesized using the HEBM technique at ambient temperature and pressure. The detail experimental conditions for the synthesis of BP powder by HEBM can be found elsewhere (see the Supplementary data)^23^. The wafer-scale of single layer graphene was synthesized by chemical vapor deposition (CVD)^20^. The sensor chip (graphene chip) with patterned graphene between the two gold (Au)-electrodes (distance of 100 µm) was fabricated in wafer-scale level by a dry-etching technique and conventional MEMS process (see the Supplementary data). The BP powder dispersed in dimethyl sulphonate (DMSO, Sigma-Aldrich) by an ultrasonic treatment was deposited on a graphene chip to fabricate a humidity sensor via an electrospray system. DMSO was used as the solvent for BP in the electrospray experiment, where BP was deposited on the patterned graphene surface. The water flow rate was controlled precisely by a syringe pump (KD 200; KD scientific). The electric potential was adjusted using a power supply (+ 0–30 kV; Korea switching) connected to the metal capillary. The metal capillary was used as an electrospray emitter with an inner diameter of 250 μm. The nozzle tip- to-substrate distance was fixed to 10 mm. For rapid evaporation of the solvent, the surface temperature of the substrate was adjusted using a hot plate (more details, see the Supplementary data). For comparison, the humidity sensor using pure BP powder was fabricated but without graphene between the Au electrodes. Humidity sensors using pure BP powder for comparison were fabricated under the same conditions but without graphene between the Au electrodes. In the wire-bonding process, the humidity sensor was mounted on the TO-39 chip to finish the sensor fabrication process.

The surfaces of the BP/graphene hybrid were characterized by field emission scanning electron microscope (FESEM, a JSM-6500F). Transmission electron microscopy (TEM) and high resolution TEM (HRTEM) images of the BP were captured using an ultra-high resolution field emission electron microscope (JEOL JEM-2100). The crystalline characteristics of the BP were investigated by X-ray diffraction (XRD, D/MAX 2500 Rigaku, Japan). Raman spectroscopy(Xplora Horiba, France) was performed in a back scattering configuration using a 532 nm laser source and holographic grating of 1200 grooves/mm.

The sensors were mounted inside an enclosed environmental chamber and a multi-meter (Fluke 8846 A) connected to the computer was used to record the resistance of the sensors. The humidity in gas chamber was generated using a water bubble controller and introduced into the chamber by N_2_ gas. A computerized mass flow controller (ATOVAC, GMC 1200) was used to change the humidity and concentration of N_2_ gas. The relative humidity (RH%) was varied from 15 to 70%. N_2_ gas with different humidity level was delivered to the chamber at a constant flow rate of 100 standard cubic centimeters per minute (sccm). The gas chamber was purged with N_2_ gas between each humidity pulse, allowing the surfaces of the sensors to return to the standby state.

## Electronic supplementary material

Supplementary Info
