# Supplementary material for: Black P/graphene hybrid: A fast response humidity sensor with good reversibility and stability
Source: Sci Rep. 2017 Sep 5;7:10561. doi: 10.1038/s41598-017-10848-3 (PMC5585363; doi:10.1038/s41598-017-10848-3)
Supplement: Supplementary file 1 — Supplementary Info [file 41598_2017_10848_MOESM1_ESM.doc]

**Supplementary Information**

**Black P/graphene hybrid: A fast response humidity sensor with good reversibility and stability**

Duy-Thach Phan^1^, Inyong Park^1^, Ah-Ram Park^2^, Cheol-Min Park^2,*^, Ki-Joon Jeon^1,*^

*^1^Department of Environmental Engineering, Inha University, 100 Inha-ro, Nam-gu, Incheon, 22212, Republic of Korea*

*^2^School of Materials Science and Engineering, Kumoh National Institute of Technology, Gumi, Gyeongbuk, 39177, Republic of Korea*

*Co-corresponding authors: [cmpark@kumoh.ac.kr](mailto:cmpark@kumoh.ac.kr)/[kjjeon@inha.ac.kr](mailto:kjjeon@inha.ac.kr)

**1. Black P synthesis**

Black P powder was synthesized using a high energy mechanical ball milling technique at ambient temperature and pressure. Red P powder (High Purity Chemicals, >99%) and stainless steel balls (diameter: 3/8 and 3/16 in.) were placed into a hardened steel vial with a capacity of 80 cm^3^ with a ball to powder ratio of 20:1, and the HEMM process was conducted under an Ar atmosphere for 12 hours.


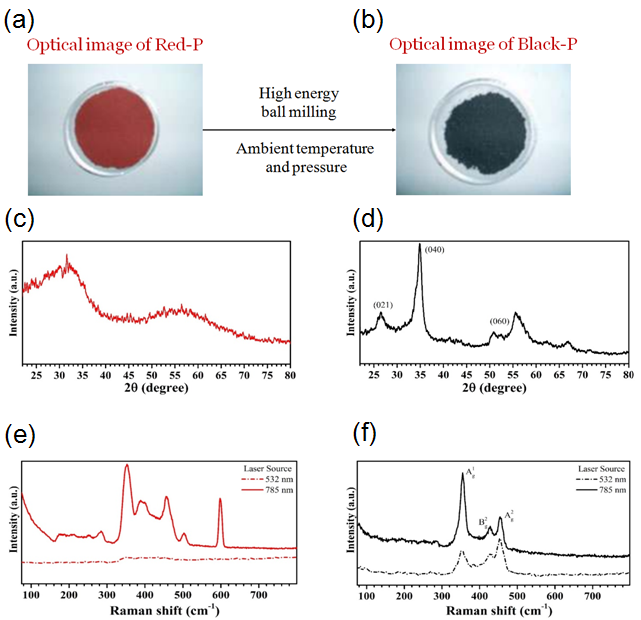


**Figure S1. Optical images of the powder form of (a) RedP before the HEBM process, (b) BlackP after HEMM process, XRD pattern of (c) RedP, (d) BlackP and Raman spectra of (e) RedP, (f) BlackP.**

**2. Sensor fabrication process**

Large scale (wafer level) graphene was synthesized on 25 μm thickness of copper (Cu) foil (Alfa Aesar, 46365) from a mixture of hydrogen (H_2_, high purity 99.999%, 25 sccm) and methane (CH_4_, high purity 99.999%, 100 sccm) at 1020 ^o^C using a chemical vapor deposition (CVD) method (Thermal CVD, Thermo Scientific). Before graphene deposition, the Cu foil was cleaned by sequence of solution (Acetone, DI water, HCl (10%), Acetone, Isopropyl Alcohol) and dry by nitrogen (N_2_) gas. The graphene on copper (Cu) foil was spin coated with poly-methyl methacrylate (PMMA) layer as a supporting layer and the Cu foil was then removed by wet etching in an aqueous solution of ammonium per sulfate (APS). Once the Cu foils were etched completely, the graphene/PMMA was rinsed in DI water for 30 min. Finally, the graphene/PMMA was transferred onto a SiO_2_/Si wafer and dried at 140 ^o^C for 30 minutes, followed by removing the PMMA coating with acetone. The patterned graphene was fabricated via a dry etching process by oxygen plasma and gold electrodes were then deposited by e-beam lithography.


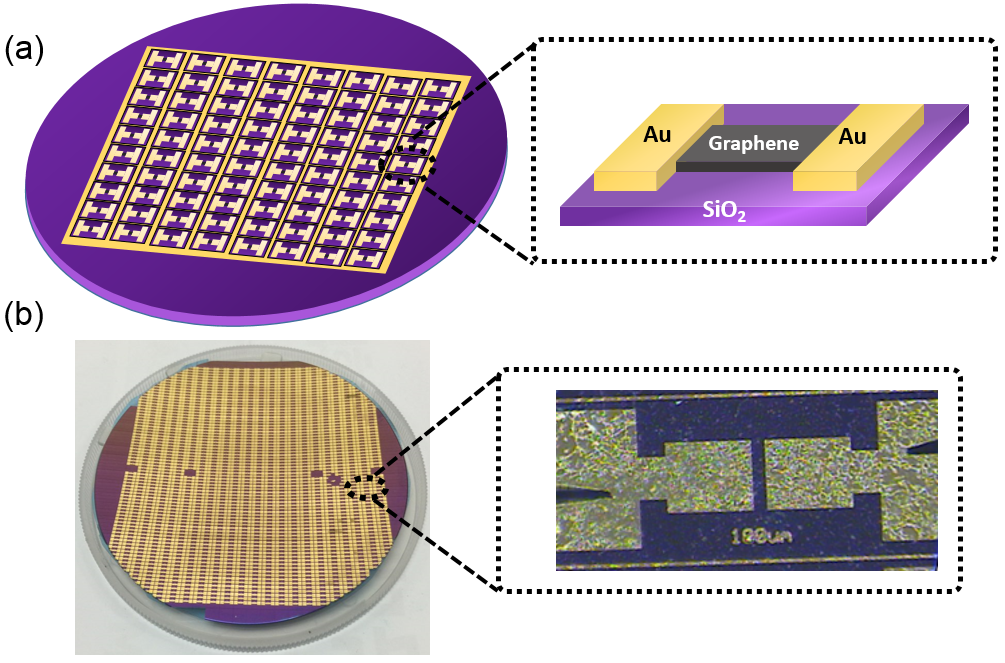


**Figure S2. Wafer-scale level fabrication of sensor (a) schematic diagram and (b) image of the sensor array on the wafer after the fabrication process.**

**3. Electrospray system**

Figure S3-1 show a schematic of the electrospray system. In this experiment, dimethyl sulfonate (DMSO, Sigma-Aldrich) was used as the solvent of black P (BP). The concentration of BP solution was 0.1 mg/ml. The 50 ml BP solution was sonicated by ultrasonic bath (150W, model-8210, Branson) for 30 minutes. The working fluid was loaded in plastic syringe (Norm JECT, HSW) and flow rates of the working fluid were precisely adjusted using a syringe pump (KD 100, KD scientific). The electric field strength was controlled by a high voltage power supply (0-+30kV, Korea switching). Stainless steel tubing was used as an electrospray nozzle (I.D.:250µm, O.D.:1.5mm). The distance between the tip and collector was maintained at 10mm in all experimental cases. The spray time of BP was 10 minutes. For the rapid evaporation of the solvent, the surface temperature of the substrate was controlled by a hot plate.


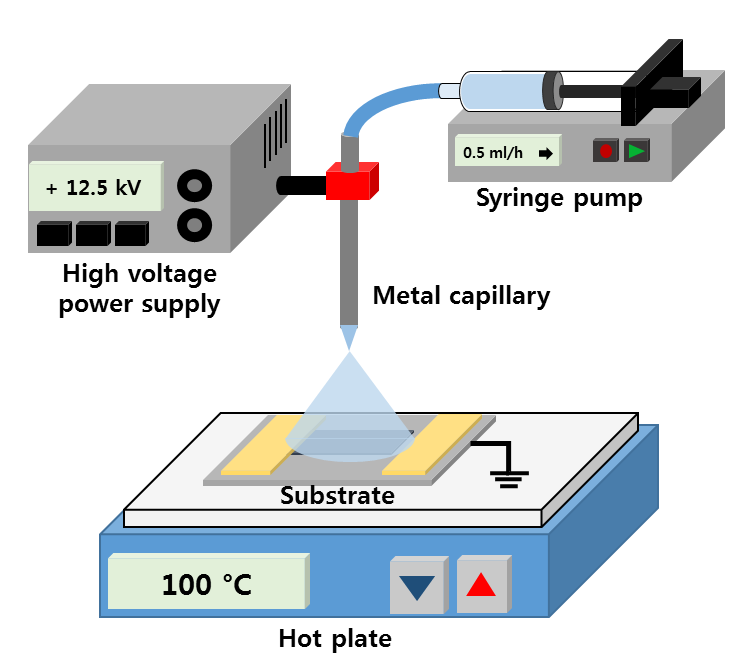


**Figure S3-1. Schematics of the electrospray system**

Figure S3-2 shows the functioning domain of a mixture of BP and DMSO. The main functioning modes of the electrospray consist of three modes, dripping, cone-jet and multi-jet mode. Figure S3-3 presents representative images of each mode. In dripping mode, the droplets are releases periodically and the size of the droplet is comparable to the size of the nozzle. As the applied voltage increases, the conical meniscus and spray plume forms at the tip of the nozzle. The formation of a conical meniscus and thin jet is visible evidence of the presence of a cone-jet mode. The cone-jet mode is the most essential mode in the electrospray technique due to the reproducibility of the finest droplet, continuously. In this system, the presence of cone-jet mode was confirmed over a wide range of flow rate conditions (0-1.5 ml/h). As the applied voltage was increased further, the tilted jet formed and the orientation of the jet irregularly changed along the circumference of the nozzle.


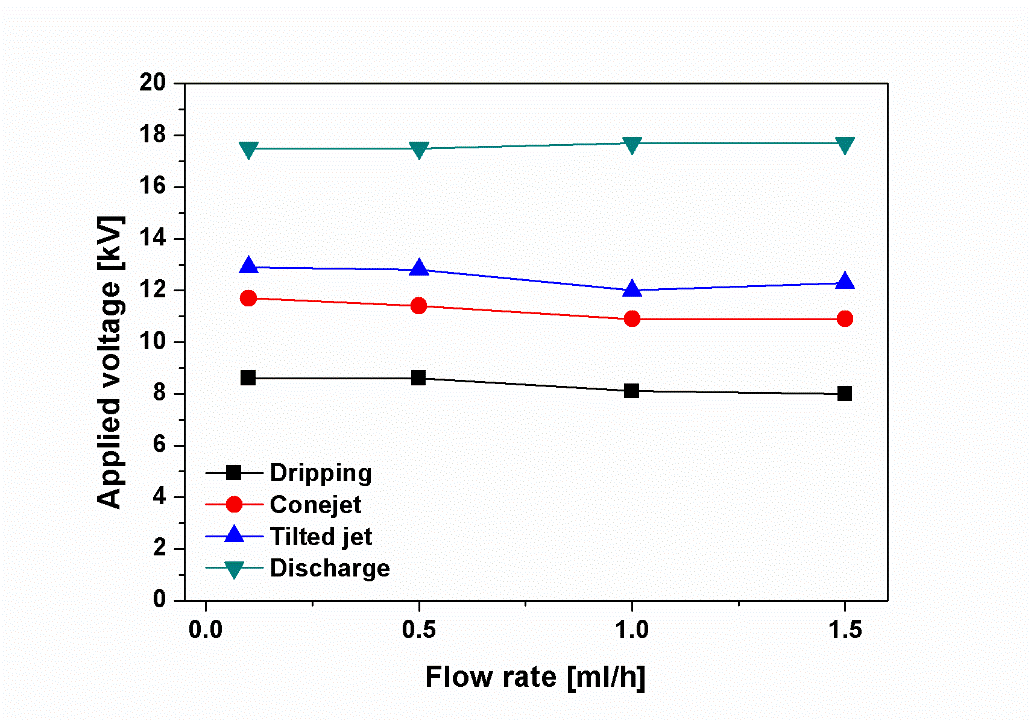


**Figure S3-2. Functioning domains of the electrospray system (working fluid: BP and DMSO)**


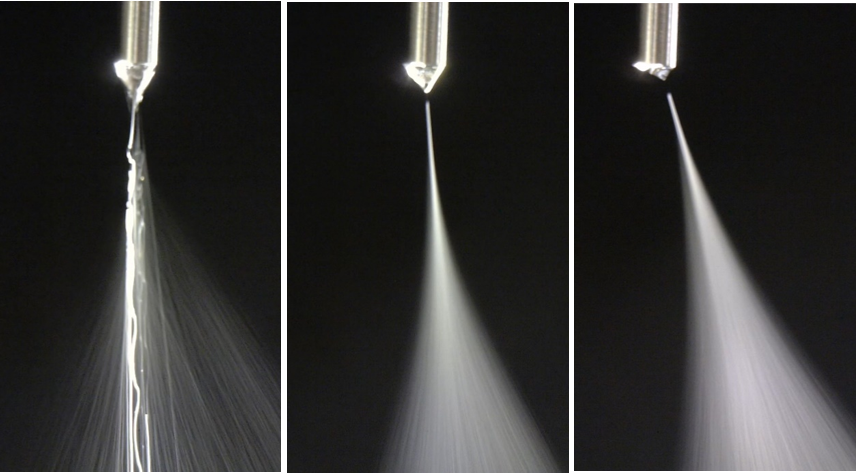


**Figure S3-3. Representative images of the functioning modes of electrospray with BP and DMSO, dripping, cone-jet, and tilted jet mode.**

**4. Raman spectra of SLG before and after fabricating sensor**


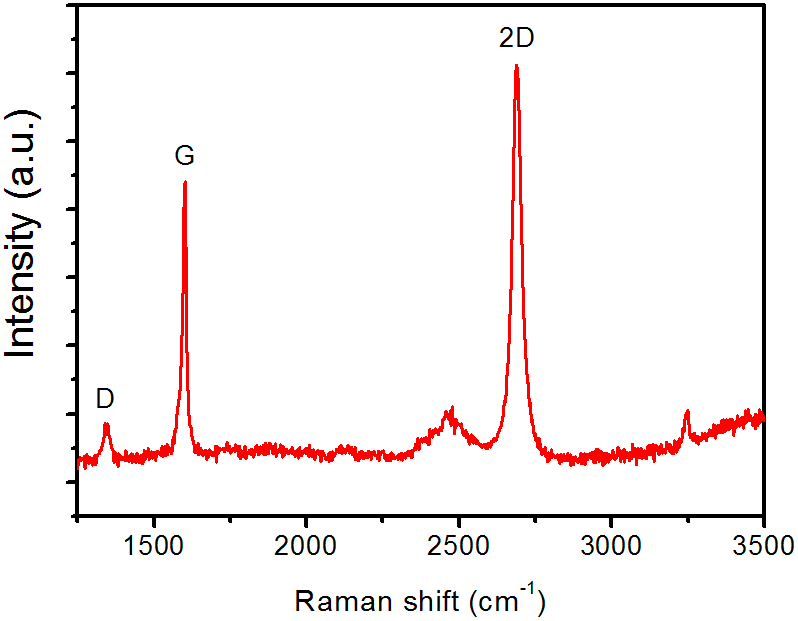


(a)


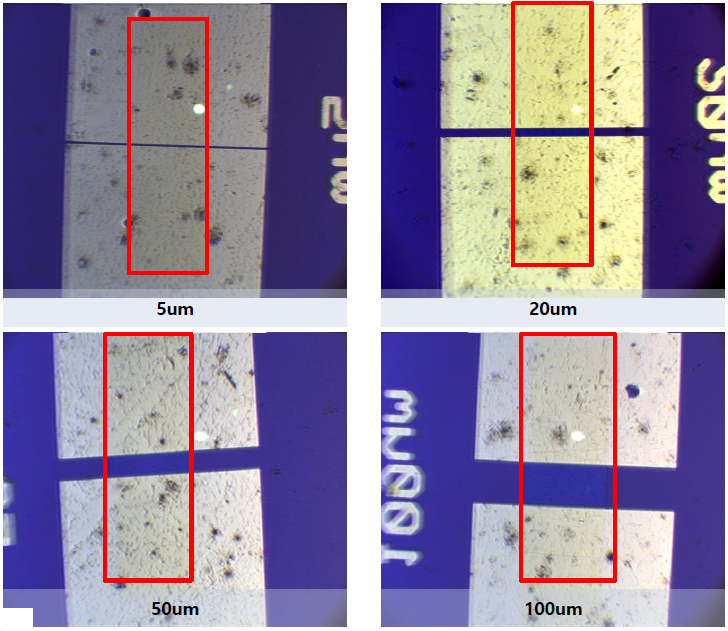


(b)

(c)

**Figure S4. (a) Raman spectra of as-synthesized SLG, (b) Optical image of fabricated graphene device with different electrode distance (before depositing BP), (c) G and 2D peak of Raman spectra of graphene (with 100 μm distance) after fabricating device (10 samples from A1 to A10).**

**5. Response of humidity sensor using pure graphene**


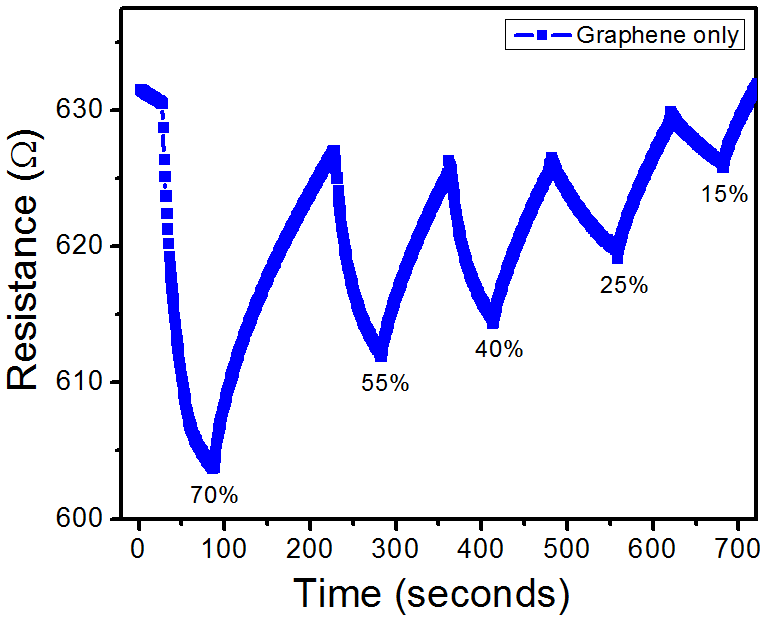


**Figure S5. The response of as-fabricated humidity sensor using pure graphene to various level of relative humidity.**

**6. Stability of humidity sensor**

Figure S6 presents the response of the humidity sensor made of BP/graphene heterojunction to 70% RH after 2 and 4 weeks. The sensor based on the BP/graphene heterojunction lost its excellent humidity sensing properties after 4 weeks.


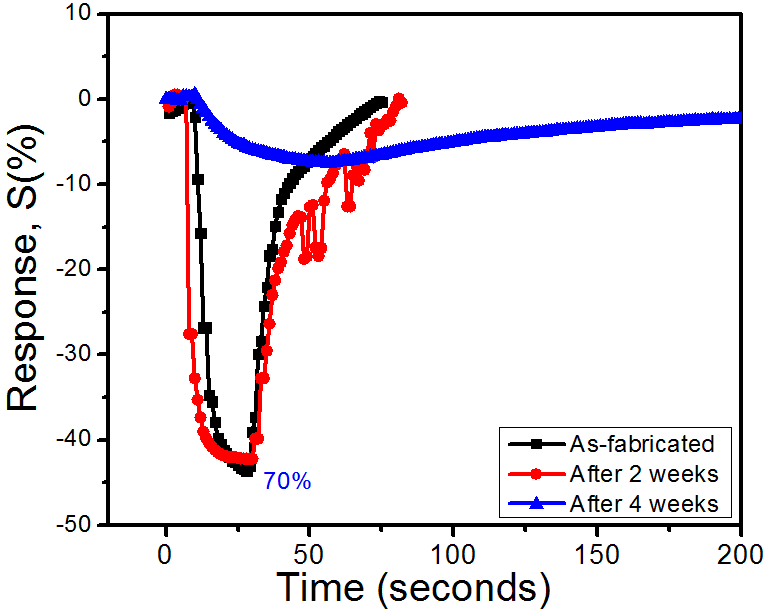


**Figure S6. Long time stability of the humidity sensor**
